# Task-specific information outperforms surveillance-style big data in predictive analytics

**DOI:** 10.1073/pnas.2020258118

**Published:** 2021-03-31

**Authors:** Andreas Bjerre-Nielsen, Valentin Kassarnig, David Dreyer Lassen, Sune Lehmann

**Affiliations:** ^a^Department of Economics, University of Copenhagen, 1353 Copenhagen, Denmark;; ^b^Center for Social Data Science, University of Copenhagen, 1353 Copenhagen, Denmark;; ^c^Institute of Software Technology, Graz University of Technology, 8010 Graz, Austria;; ^d^Center for Economic Behavior and Inequality, University of Copenhagen, 1353 Copenhagen, Denmark;; ^e^DTU Compute, Technical University of Denmark, 2800 Kongens Lyngby, Denmark

**Keywords:** academic performance, prediction, big data, privacy

## Abstract

Increasingly, human behavior can be monitored through the collection of data from digital devices revealing information on behaviors and locations. In the context of higher education, a growing number of schools and universities collect data on their students with the purpose of assessing or predicting behaviors and academic performance, and the COVID-19–induced move to online education dramatically increases what can be accumulated in this way, raising concerns about students’ privacy. We focus on academic performance and ask whether predictive performance for a given dataset can be achieved with less privacy-invasive, but more task-specific, data. We draw on a unique dataset on a large student population containing both highly detailed measures of behavior and personality and high-quality third-party reported individual-level administrative data. We find that models estimated using the big behavioral data are indeed able to accurately predict academic performance out of sample. However, models using only low-dimensional and arguably less privacy-invasive administrative data perform considerably better and, importantly, do not improve when we add the high-resolution, privacy-invasive behavioral data. We argue that combining big behavioral data with “ground truth” administrative registry data can ideally allow the identification of privacy-preserving task-specific features that can be employed instead of current indiscriminate troves of behavioral data, with better privacy and better prediction resulting.

In the field of higher education, the application of (mostly) digital data on student behavior in and out of classrooms for predictive purposes is known as *learning analytics* or *educational data mining*. The key premise of learning analytics is that pervasive data collection and analysis allows for informative predictions about academic behavior and outcomes (1), although potentially at the cost of student privacy and agency (2), as highlighted in recent media coverage (3, 4). Such concerns have most recently been amplified with the massive shift toward online education following the COVID-19 pandemic (5–7).

Learning analytics, and, more generally, data analytics, employ as inputs the digital traces that people leave behind when going about their daily business (8), searching the internet (9), using smartphones (10, 11), and engaging on social media (12). Such traces—known also as digital breadcrumbs (13) or behavioral surplus (14)—are increasingly valued for their role in predicting behavior both in the market place and in public sector settings, and, increasingly, in higher education.

Digital traces of student behavior at the individual level—for example, interaction with digital portals, WiFi use, and location-based services—can be used to predict key student outcomes such as academic performance and dropout (15–17). However, while such “big data” may have predictive power, the benefits of this come at a potential cost of loss of privacy (14).

The *privacy*–*utility tradeoff* (18–20) posits that predictive ability from personal data is inversely related to privacy preservation. While generally true within a given dataset, this approach neglects the possibility that other data, possibly from different sources, on the same set of individuals may have a superior predictive ability for a given, or even more favorable, level of privacy. We argue—following the logic of prediction contests, where new candidate models are compared against the best possible alternative rather than a benchmark of zero predictive ability—that we should compare the predictive ability of different datasets, with different levels of granularity and potential privacy implications, to make more-informed choices about prediction/privacy tradeoffs. This insight is particularly important for characteristics or behaviors that are more stable over time and for outcomes where past task-specific information is available.

One outcome where information on past task-specific activities is available is school grades: Students in higher education have taken multiple examinations prior to college enrollment, and the results of these are highly informative about future performance. In our sample of engineering college students, using the administrative registry data described below, 68.6% of students with a medium-level high school grade point average (GPA) also place in the medium GPA range in college. This suggests the possibility that predicting academic performance may not require knowledge of sensitive information from browser use or smartphones, but simply a measure of past performance often used as entry criteria in higher education in the first place.

## Approach

To examine the relative predictive performance of granular “big data” and summary measures of past performance, we combine detailed big data on behavior and personality from the Copenhagen Network Study (11, 21) with administrative data collected by official sources for research purposes, recognized for their high quality (22). The ability to follow students across both big and administrative datasets is unique to this study, as granular big data on behavior is typically proprietary to corporate data collectors, who do not have access to high-quality survey or administrative data, and instead predict traits and characteristics using their behavioral datasets (23, 24). Our big data consist of individual measures of campus behavior and social network position as well as personality directly measured from surveys but often inferred from social media data (23), collected across 2 y for 521 individuals; see Table 1 for etails. In our setting, administrative data were collected prior to enrollment, and big data were collected after.

**Table 1.** Feature sets

Administrative data	Big data
Sociodemographic background	Behavior
Age	Class attendance
Gender	In-class smartphone use
Immigration	Time on campus
Education of parents	Mean GPA of contacts^†^
Income of parents	Degree centrality^†^
Wealth of parents	Personality
Past performance	Alcohol consumption
High school GPA	Ambition
High school grades in	Big Five Index (OCEAN)
(Math, Language*)	Homophily academic
Middle school grades in	performance
(Math, Language*)	Locus of control
	Physical activity
	Self-efficacy
	Self-rated academic
	performance (now, past)

* Average of grades in Danish and English.

^†^ Average of grades in Danish and English.

### Big Data vs. Administrative Data.

To examine performance, we build three sets of predictive models of students’ academic performance, measured as cumulative GPA in June 2015, by using the two data sources separately, in combination, and on specific subsets of the data. In each model, the goal is to classify students into categories of low, medium, or high performance, defined as the bottom 20%, middle 60%, and top 20%, respectively. We use logistic regression methods to classify the students, as these yield the best predictive performance, but other methods, such as random forest prediction, have similar outcomes.

## Results

**Fig. 1.**
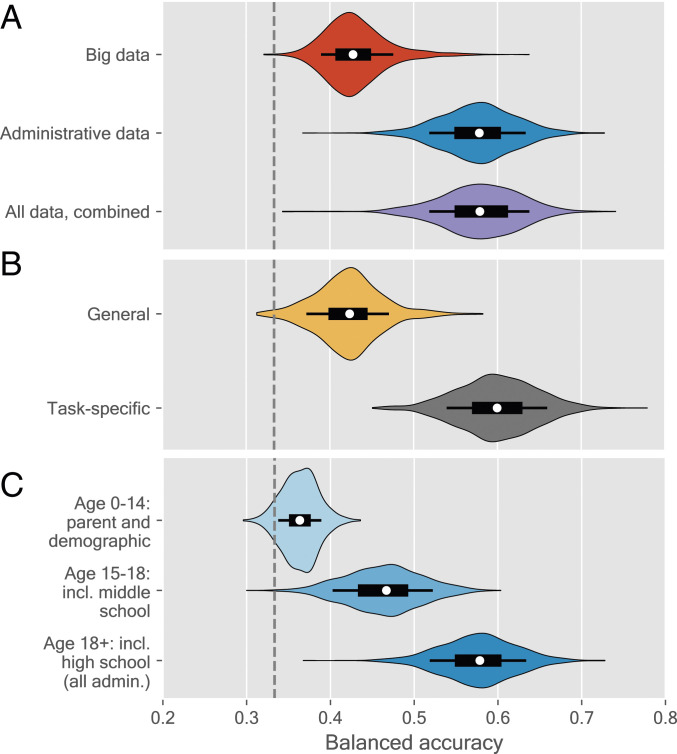
Balanced accuracy of model out of sample on test data when using various feature sets. (*A*) Big data vs. administrative data, (*B*) task-related vs. general information, and (*C*) comparison of feature sets gathered over the lifespan of the student. Models are estimated using logistic regression with $L_{2}$ regularization and using feature selection; see *Materials and Methods* for details. Each violin represents the distribution of weighted accuracy from 1,000 resamples. Inside the violins, the thick bar represents the bottom and top quartiles, and the thin lines represent the bottom and top deciles. The dashed, black line indicates the performance of a baseline random guessing model.

Fig. 1*A* shows the basic comparisons. The top entry shows results from the big behavioral dataset using violin plots (25), where we find a balanced accuracy of 43%, significantly higher than the random guess with balanced accuracy of 33%. In contrast, using historical administrative data only (middle entry), we find a mean balanced accuracy of 58%, 15 percentage points higher than the model based on detailed behavioral data (p<0.0001 from a follow-up corrected resampled t test).

Combining the two data sources to investigate whether behavioral data can add predictive value to administrative data yields a model (Fig. 1*A*, bottom entry) not significantly different from the model based on administrative data (p=0.910). This lack of improvement when incorporating the behavioral data from 2 y of college suggests that, in the present sample, academic performance is largely stable after graduating from high school and that predictive ability is not improved by detailed digital trace data.

### “All Big Data” vs. the “Right” Data.

The terms *digital breadcrumbs* and *behavioral surplus* suggest an indiscriminate “big” data collection of identifiable scraps, often collected as by-products of other activities and at low cost, with minimal or no consent, but with potentially large privacy costs per unit of prediction accuracy. In contrast, task-specific data focus on the prediction problem at hand and are, arguably, less privacy invasive than high-granular data.

In order to explore the hypothesis that task-specific data improve prediction, we now distinguish predictive models from two disjointed sets of features: task-specific and general information. Task-specific information contains all measures of historical educational performance (middle and high school grades), while general information contains everything else, both administrative and big data. Fig. 1*B* shows the predictive performance: The task-specific model (bottom entry) clearly outperforms the general information model (top entry), with the task-specific model gaining 18 percentage points in accuracy on the general information model (p<0.0001). Even with the very rich measurements available regarding individual students, measures of past educational performance are superior predictors for future educational performance.

### Development of Prediction across the Lifespan.

While school outcome trajectories are relatively stable across time, university academic outcomes are well predicted neither from socioeconomic status (SES) alone nor from adding middle school outcomes. Fig. 1*C* shows that including all SES data available at age 0 y to 14 y has little predictive power and performs only marginally better than the baseline (and worse than the big data; Fig. 1*A*), and adding middle school grades and SES until age 18 y yields an approximately 10 percentage point gain in terms of accuracy (p<0.001), now outperforming postenrollment data. Finally, including all preenrollment data (ages 18+ y), we observe yet another 10 percentage point gain (p<0.001), recovering our performance from Fig. 1 *A* and *B*.

## Discussion

In recent years, educational institutions have started systematically collecting and analyzing digital data about their students, often in the form of digital breadcrumbs from digital educational services or WiFi use, with the aim of improving understanding and prediction student behavior. The COVID-19 pandemic is projected to dramatically accelerate this process, intensifying already existing worries about privacy and transparency, as well as broader ethical and legal concerns (2, 26, 27), over the collection of such high-resolution data.

The results presented here indicate that big behavioral data do indeed have predictive value (noting that analyses of such data are sensitive to choices of, e.g., data cleaning and methods). Importantly, however, we find that much narrower task-specific data on historical outcomes result in better predictions at lower costs to privacy. Therefore, we propose that privacy-invasive data collection should always be compared against other possible data sources, potentially with different privacy properties. Moreover, complex prediction models suffer, in addition to well-known problems of population inference and increased variability from convenience sampling (28), from lack of transparency and, related to this, risk of bias; in our case, a random forest model employing complex big data with minimal interpretability is outperformed by an easily interpretable logit model with task-specific information on past grades. As prediction models are increasingly used across multiple domains, it becomes ever more important to assess the performance of models employing privacy-invasive data against simpler, sparser models informed by domain expertise that can provide the same or better predictive performance at lower privacy costs.

We note that our study is confined to students who participated in the study among first- and second-year cohorts from a single technical university in Denmark, with limited variation in age as well as cultural and socioeconomic background. Further studies in other educational and cultural contexts will be important for a fuller examination of tradeoffs between predictive ability, privacy, and task-specific information.

## Materials and Methods

### Data.

The full study protocol was approved by the Danish Data Supervision Authority. Dataset 1 is administrative registry data from Statistics Denmark. It consists of sociodemographic and education data; see “Administrative data” in Table 1. Dataset 2 is data from the Copenhagen Network Study (6, 21), which includes measures of behavior and personality after enrollment; see “Big data” in Table 1. From the latter, we extracted features representing the students’ behavior and their social network. The feature extraction process is described in ref. 29. For computation of class attendance and screen time, see refs. 16 and 17.

### Machine Learning Approach.

We built classification models to predict the student GPA category and tested the model on randomly selected holdout data to measure its accuracy without bias from overfitting. To ensure that our conclusions incorporated modeling uncertainty, we repeated the estimation process 1,000 times using nested resampling (30). For each split, we reserved 75% of observations for the training phase, that is, model estimation and calibration, with the remaining 25% used for testing. In the training phase, we used two repetitions of fivefold cross-validation to select the hyperparameters (i.e., K, $\lambda^{pre}$, and $\lambda^{post}$) that yielded lowest cross-entry on the validation sets. Each model was estimated according to the following steps: 1) Replace missing values using the mean along each column; 2) scale all features into zero mean, unit standard deviation; 3) select the K best performing features using a logistic regression where $L_{2}$ regularization is set to $\lambda^{pre}$; and 4) estimate the model again using a logistic regression where $L_{2}$ regularization is set to $\lambda^{post}$. We compared the model performance in terms of the distributions resulting from the nested resampling procedure. To measure the statistical difference of model performance, we use the corrected resampled *t* test statistic (31). Our results are robust to using equal size grade categories and to the number of categories.

## Data Availability

Anonymized full code and the (anonymized) model output data used to construct the figures in the paper have been deposited in GitHub (https://github.com/SocialComplexityLab/big_vs_right_data) (32).
